# Intrahepatic microcirculatory disorder, parenchymal hypoxia and NOX4 upregulation result in zonal differences in hepatocyte apoptosis following lipopolysaccharide- and D-galactosamine-induced acute liver failure in rats

**DOI:** 10.3892/ijmm.2013.1573

**Published:** 2013-12-03

**Authors:** MASATAKE TANAKA, KOSUKE TANAKA, YUKO MASAKI, MASAYUKI MIYAZAKI, MASAKI KATO, KAZUHIRO KOTOH, MUNECHIKA ENJOJI, MAKOTO NAKAMUTA, RYOICHI TAKAYANAGI

**Affiliations:** 1 Department of Medicine and Bioregulatory Science, Graduate School of Medical Sciences, Kyushu University, Fukuoka 812-8582, Japan; 2 Faculty of Pharmaceutical Sciences, Fukuoka University, Fukuoka 814-0180, Japan; 3 Department of Gastroenterology, Kyushu Medical Center, National Hospital Organization, Fukuoka 810-8563, Japan

**Keywords:** acute liver failure, macrophage, hypoxia, oxidative stress, microcirculation, apoptosis, nicotinamide adenine dinucleotide phosphate oxidase, pimonidazole, cleaved caspase-3, 4-hydroxy-2-nonenal, reactive oxygen species, hypoxia-inducible factor 1-α

## Abstract

Although the mechanisms responsible for acute liver failure (ALF) have not yet been fully elucidated, studies have indicated that intrahepatic macrophage activation plays an important role in the pathogenesis of ALF through intrahepatic microcirculatory disorder and consequent parenchymal cell death. Intrahepatic microcirculatory disorder has been demonstrated in animal models using intravital microscopy; however, the limitations of this method include simultaneously evaluating blood flow and the surrounding pathological changes. Therefore, in this study, we devised a novel method involving tetramethylrhodamine isothiocyanate (TRITC)-dextran administration for the pathological assessment of hepatic microcirculation. In addition, we aimed to elucidate the mechanisms through which intrahepatic microcirculatory disorder progresses with relation to activated macrophages. ALF was induced in Wistar rats by exposure to lipopolysaccharide and D-galactosamine. Intrahepatic microcirculation and microcirculatory disorder in zone 3 (pericentral zone) of the livers of rats with ALF was observed. Immunohistochemical examinations in conjunction with TRITC-dextran images revealed that the macrophages were mainly distributed in zone 2 (intermediate zone), while cleaved caspase-3-positive hepatocytes, pimonidazole and hypoxia-inducible factor 1-α were abundant in zone 3. We also found that 4-hydroxy-2-nonenal and nicotinamide adenine dinucleotide phosphate oxidase (NOX)4-positive cells were predominantly located in the zone 3 parenchyma. The majority of apoptotic hepatocytes in zone 3 were co-localized with NOX4. Our results revealed that the apoptotic cells in zone 3 were a result of hypoxic conditions induced by intrahepatic microcirculatory disorder, and were not induced by activated macrophages. The increased levels of oxidative stress in zone 3 may contribute to the progression of hepatocyte apoptosis.

## Introduction

Acute liver failure (ALF) is a syndrome defined by the sudden onset of severe liver injury, followed by coagulopathy and encephalopathy. The mortality rates for patients with ALF are 50–70% (1–3). The prognosis of ALF has not improved significantly over the past decades, despite the development of treatments, such as plasma exchange, dialysis and antibiotics. Although liver transplantation is the only promising treatment for ALF, the rapid progression of the disease and the shortage of donors limit the success of this treatment option (3). However, the pathogenesis of ALF is not yet fully understood and therefore prohibits us from establishing new treatments.

It has been suggested that intrahepatic microcirculatory disorder is involved in the pathogenesis of ALF. Intrahepatic macrophage activation has been suggested as an important factor in the pathogenesis of ALF, causing intrahepatic microcirculatory disorder, and subsequently, massive levels of hepatocyte death (4,5). Verification that activated macrophages disturb intrahepatic microcirculation has not been achieved due to the difficulties in visualizing the changes occurring in macrophages and microcapillary structure simultaneously.

Intravital microscopy, used for the observation of biological systems with high resolution, is an excellent technique due to its ability to obtain real-time dynamic microcirculatory images (6,7). It has often been used in experimental animal models of liver injury (8–11). The limitations of this method include difficulty in simultaneously evaluating blood flow and surrounding pathological changes. Furthermore, only a limited number of laboratories are able to utilize this technique as it demands expensive and specialized equipment and facilities.

In this study, we designed a method for visualizing hepatic microcapillary structure with the administration of tetramethylrhodamine isothiocyanate (TRITC)-dextran. Applying this method to lipopolysaccharide (LPS)- and D-galactosamine (GalN)-induced liver injury in rats, a widely accepted animal model of ALF (12), we were able to obtain detailed findings of intrahepatic microcirculatory disturbance in relation to intrahepatic macrophage activation. Given that the massive reduction in the number of viable hepatocytes is caused by intrahepatic microcirculatory disorder, there still remains the controversial issue of whether this reduction in ALF is due to apoptosis or necrosis. A number of studies have demonstrated that activated macrophages can cause intrahepatic microcirculatory disorder and consequent parenchymal necrosis (13–18), while others have indicated that activated macrophages induce parenchymal apoptosis by secreting death receptor ligands, such as Fas ligand or tumor necrosis factor-α (TNF-α) (19,20). Although it is difficult to determine which manner of cell death is dominant, recent studies have indicated that apoptosis plays a major role in the reduction in the number of viable hepatocytes during the development of ALF (42,43).

Apoptosis is induced through the sequential activation of caspases that can be activated by death receptors (extrinsic pathway) also cellular stress (intrinsic pathway) (21). As regards the intrinsic pathway, the nicotinamide adenine dinucleotide phosphate oxidase (NOX) family is a major source of cellular reactive oxygen species (ROS) (22–24). Among NOX homologues, only NOX4 is an inducible isoform (25). To evaluate the mechanisms that trigger hepatocyte apoptosis in ALF, we conducted an immunohistochemical detection of cleaved caspase-3, CD68, pimonidazole, hypoxia-inducible factor 1-α (HIF1-α), 4-hydroxy-2-nonenal (4-HNE) and NOX4 in rat livers, and aimed to determine correlations between their localization and intrahepatic microcirculatory disorder.

## Materials and methods

### Chemicals and antibodies

All reagents were purchased from Sigma Chemical Co. (St. Louis, MO, USA) unless otherwise stated. Monoclonal mouse anti-CD68 and rabbit anti-NOX4 antibodies, as well as horseradish peroxidase (HRP)-conjugated polyclonal goat anti-mouse and goat anti-rabbit antibodies were purchased from Abcam (Cambride, UK). The polyclonal rabbit anti-cleaved caspase-3 and rabbit anti-HIF1-α antibodies were from Novus Biologicals (Littleton, CO, USA). A monoclonal mouse anti-4-HNE antibody was obtained from NOF Corp. (Tokyo, Japan). Pimonidazole and the mouse anti-pimonidazole adducts monoclonal antibody (Hypoxyprobe™-1 kit) were purchased from Hypoxyprobe Inc. (Burlington, MA, USA).

### Animals

Eight-week-old male Wistar rats (weighing approximately 200 g), were purchased from Japan SLC, Inc. (Hamamatsu, Japan). The animals were provided access to food and water *ad libitum* and maintained on a 12-h light-dark cycle in a temperature (18–21°C) and humidity (55±5%) controlled environment for 1 week before the experiments. All animals received humane care, and all experiments in this study were performed according to the guidelines of the Committee for the Care and Use of Laboratory Animals at Kyushu University, Fukuoka, Japan.

### Experimental protocols

The rats were divided into 2 groups (n=10 rats/group). LPS from *Escherichia coli* (5 μg/kg body weight) and GalN (500 mg/kg body weight) were intraperitoneally injected into the rats in the ALF group, while sterile saline was injected into the rats in the control group (n=10). At 22 h post-administration, pimonidazole (60 mg*/*kg body weight) was intraperitoneally injected into all the rats. Pimonidazole is rapidly reduced in hypoxic cells to a highly reactive intermediate that binds to sulfur on glutathione and proteins. Using an antibody against these pimonidazole-sulfur adducts, immunohistochemistry can be employed to detect the hypoxic cells (26–33). Two hours after the pimonidazole injection, the rats were anesthetized with isoflurane, and the abdomens were incised and canulated at the inferior vena cava using a 20 G plastic needle. After a 1-ml blood sample was withdrawn, the rats were intravenously injected with TRITC-dextran (500 mg/kg body weight) dissolved in 1 ml of sterile saline and the livers excised. One lobe of excised liver was fixed in 20% formaldehyde solution (Mildform 20N; Wako Pure Chemical Industries, Osaka, Japan) for histological analysis, and another lobe was snap-frozen in liquid nitrogen for use in western blot analysis.

### Preparation of liver sections to assess TRITC-dextran distribution

The excised livers were fixed in 20% formaldehyde solution for 1 day, paraffin-embedded and sectioned (5 μm thickness). The sections were deparaffinized with xylene and rehydrated by washing through a graded alcohol series and deionized water. The hydrated tissue sections were washed with phosphate-buffered saline (PBS). To evaluate TRITC-dextran distribution, the sections were mounted in aqueous Fluor Mount/plus (Diagnostic Biosystems Inc., Pleasanton, CA, USA) immediately after deparaffinization and observed under a fluorescence microscope (BZ-9000; Keyence, Osaka, Japan).

### Hematoxylin and eosin (H&E) staining

Some sections were deparaffinized, rehydrated and stained with H&E to evaluate the general histological state. Some sections were deparaffinized, rehydrated and stained with H&E according to a standard procedure (66) to evaluate the general histological state.

### Immunohistochemistry

The liver sections were deparaffinized, rehydrated and exposed to 3% (v/v) H_2_O_2_ for 15 min at room temperature to quench endogenous peroxidase activity. The sections were incubated with antigen retrieval solution (Target Retrieval Solution; Dako, Tokyo, Japan) for 30 min at 95°C. Following 2 washes in PBS, the sections were incubated with blocking buffer (Blocking One Histo; Nakalai Tesque, Inc., Kyoto, Japan) for 30 min. The sections were then incubated with antibodies dissolved in Can Get Signal Immunostain solution A (Toyobo, Osaka, Japan) for 1 h at room temperature, followed by incubation with the appropriate secondary antibody conjugated to HRP in Can Get Signal Immunostain solution B for 30 min at room temperature. Following 2 washes in PBS, the sections were incubated with 3,3′-diaminobenzidine tetrahydrochloride (DAB) solution for 5 min.

For double immunohistochemical staining, the same steps from antigen retrieval to incubation with a chromogen described above were repeated, except that acetonitrile solution (Vector SG; Vector Laboratories, Burlingame, CA, USA) instead of DAB was used as a chromogen. The sections were washed twice with PBS and mounted in aqueous Fluor Mount*/*plus. Hematoxylin counterstaining was only conducted for the samples incubated with a single antibody. For CD68 and pimonidazole immunohistochemistry, we obtained bright-field and fluorescent images from the same liver sections and merged them. For double immunohistochemistry and HIF1-α, 4-HNE and NOX4 immunohistochemistry, staining was carried out on serial sections. The pathological findings assessed by one of the authors blinded to the group allocations.

### Western blot analysis

Liver tissue samples (20 mg) were homogenized in lysis buffer and protein levels were quantified using a commercial protein assay kit (Bio-Rad, Hercules, CA, USA). Liver extracts (30 μg protein/lane) were electrophoresed and subjected to SDS-PAGE under reducing conditions using 10% polyacrylamide gels. Proteins were transferred to Immobilon-P polyvinylidene difluoride transfer membranes (Millipore, Billerica, MA, USA) and probed with an anti-NOX4 rabbit monoclonal antibody. Detection was carried out using an anti-rabbit IgG goat HRP-conjugated antibody in conjunction with ECL Prime Western Blotting Detection reagent (GE Healthcare, UK Ltd., Buckinghamshire, UK). The quantification of enhanced chemiluminescence (ECL) was performed using an ImageQuant LAS 4000 imaging system (GE Healthcare, UK Ltd.).

### Statistical analysis

Data are expressed using box and whisker plots. The Steel test was applied to analyze results between 2 groups, and the Steel-Dwass test was used to analyze results between 3 or more groups. A P-value ≤0.05 was considered to indicate a statistically significant difference.

## Results

### Histology of livers of rats with LPS/GalN-induced ALF

**Figure 1:**
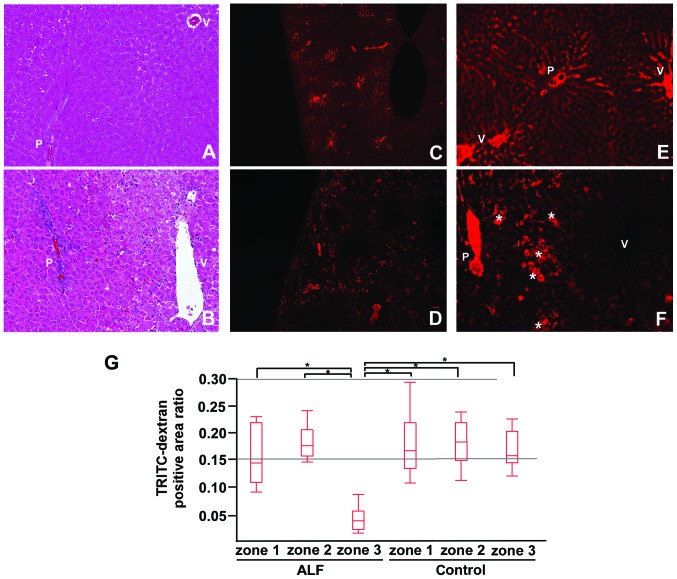
Hematoxylin and eosin (H&E)-stained sections and fluorescent images from tetramethylrhodamine isothiocyanate (TRITC)-dextran-injected livers. Liver sections from (A) control rats, and (B) rats with acute liver failure (ALF) induced by lipopolysaccharide/D-galactosamine (LPS/GalN) stained with H&E. Fluorescent images of TRITC-dextran-injected livers from (C and E) the control rats and (D and F) rats with ALF. (G) The TRITC-dextran distribution ratio for each zone in the livers from the control rats and rats with ALF. (C and E) In the livers of the control rats, TRITC-dextran was evenly distributed in the portal veins (P), sinusoids and central veins (V). (D and F) In the livers of rats with ALF, TRITC-dextran was distributed in the portal veins (P) and zone 1 sinusoids, but not in the central veins and zone 3 sinusoids. In zone 2, several spotty signals of TRITC-dextran can be obseved (*). Original magnification, ×200 (A, B, E and F), and ×40 (C and D). The TRITC-dextran distribution ratio in zone 3 of the livers of rats with ALF was significantly decreased compared with ALF zones 1 and 2, and zones 1–3 in the controls (^*^P≤0.05). Zone 1, periportal zone; zone 2, intermediate zone; zone 3, pericentral zone.

The liver sections obtained from the control rats had an integrated structure of hepatic lobules (Fig. 1A). The intraperitoneal injection of LPS/GalN induced marked hepatic injuries, accompanied by hemorrhaging and inflammatory cell infiltration (Fig. 1B).

### TRITC-dextran distribution in liver sections

We varied the interval from TRITC-dextran injection to liver excision (30 sec, 1, 2, 3, 5 and 10 min), and concluded that clear and strong signals of TRITC-dextran could be obtained after 1 min (data not shown). The interval was therefore fixed to 1 min in subsequent experiments. In the liver sections from the control rats, TRITC-dextran was distributed evenly across the liver acinus (portal veins, sinusoids and central veins) (Fig. 1C and E). In the liver sections from the rats with ALF, TRITC-dextran was clearly distributed in the portal veins and zone 1 (periportal zone) sinusoids, and could be faintly observed in the central veins and zone 3 (pericentral zone) sinusoids. Additionally, several spotty signals corresponding to TRITC-dextran appeared in zone 2 (intermediate zone) (Fig. 1D and F). We measured the ratio of TRITC-dextran-positive areas to the parenchymal area in each zone (Fig. 1G). TRITC-dextran distribution in zone 3 of the livers of rats with ALF was markedly decreased when compared with that in zones 1 and 2 of the livers of rats wtih ALF, and all zones in the livers of the control rats.

### Correlation between intrahepatic microcirculation and infiltration of CD68^+^ positive cells

**Figure 2:**
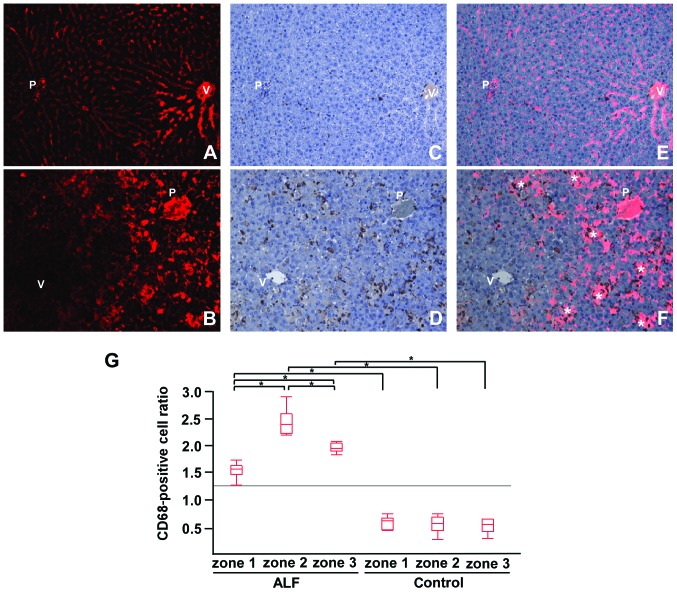
Tetramethylrhodamine isothiocyanate (TRITC)-dextran and CD68 immunohistochemistry. (A and B) TRITC-dextran distribution. (C and D) Immunohistochemical detection of CD68 and (E and F) merge images for controls (upper panel) and acute liver failure (ALF) (lower panel) samples. (G) The CD68^+^ cell ratio in each zone for the control and ALF samples. In the controls, TRITC-dextran was evenly distributed in the liver acinus (A) with some CD68^+^ cells (C). In ALF livers, marked infiltration of CD68^+^ was observed in the liver acinus (D). Merge images (B and D) showed that CD68^+^ cells were predominantly localized around the spotty signals of TRITC-dextran in zone 2 (*) (F) (original magnification, ×200). CD68^+^ cells in the ALF samples were significantly increased compared with the controls. (G) In the ALF samples, CD68^+^ cells were most abundant in zone 2, followed by zone 3 and then zone 1 (^*^P≤0.05). P, portal vein; V, central vein. Zone 1, periportal zone; zone 2, intermediate zone; zone 3, pericentral zone.

In the control rats, only some cells were positive for CD68 (Fig. 2C). In the rats with ALF, there was a marked infiltration of CD68^+^ cells distributed among the liver acinus (Fig. 2D). We observed that CD68^+^ cells dominantly localized around the spotty signals of TRITC-dextran in zone 2 (Fig. 2F). The ratio of CD68^+^ cells to the parenchymal area in each zone indicated that the CD68^+^ cells in all zones in the livers of rats with ALF were significantly increased in comparison to those in the control rats; the CD68^+^ cells were most abundant in zone 2 in the livers of rats with ALF (Fig. 2G).

### Apoptotic hepatocytes and their localization

**Figure 3:**
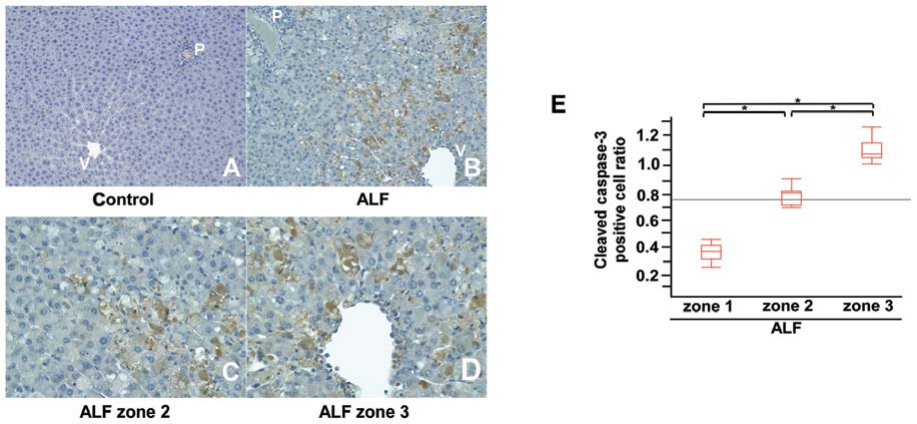
Immunohistochemical detection of cleaved caspase-3 and the apoptotic hepatocyte ratio. Immunohistochemistry for cleaved caspase-3 in livers from (A) control rats and (B–D) rats with acute liver failure (ALF). (E) The cleaved caspase-3^+^ cell ratio in each ALF zone. (A) In the controls, few cells were positive for cleaved caspase-3. (B) In the livers of mice with ALF, high numbers of cleaved caspase-3^+^ hepatocytes were observed, particularly in zones 2 (C) and 3 (D). Original magnification, ×200 (A and B), ×400 (C and D). Cleaved caspase-3^+^ cell ratios in the livers of rats with ALF were greatest in zone 3, followed by zones 2 and 1 (E) (^*^P≤0.05). P, portal vein; V, central vein. Zone 1, periportal zone; zone 2, intermediate zone; zone 3, pericentral zone.

In the control rats, a few cells were positive for cleaved caspase-3 (Fig. 3A). In the rats with ALF, high numbers of cleaved caspase-3^+^ hepatocytes were observed (Fig. 3B) and were mainly localized in zones 2 and 3 (Fig. 3C and D). In the rats with ALF, we evaluated the ratio of cleaved caspase-3^+^ hepatocytes to the parenchymal area in each zone (Fig. 3E). Cleaved caspase-3^+^ hepatocytes were most abundant in zone 3.

### Localization of parenchymal hypoxia, HIF1-α, oxidative stress and NOX4

**Figure 4:**
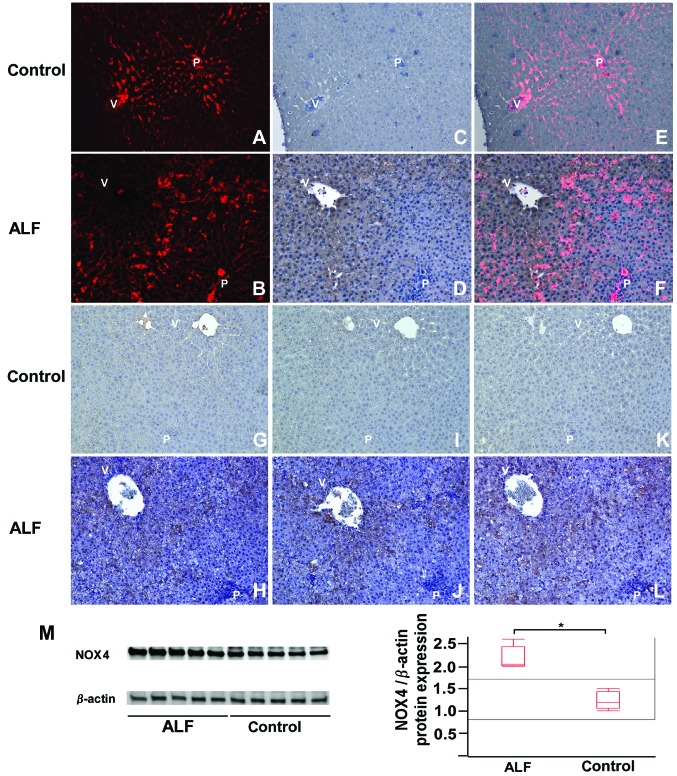
Immunohistochemical detection of pimonidazole, hypoxia inducible factor 1-α (HIF1-α), 4-hydroxy-2-nonenal (4-HNE) and nicotinamide adenine dinucleotide phosphate oxidase (NOX4), and NOX4 western blot analysis results. (A and B) Tetramethylrhodamine isothiocyanate (TRITC)-dextran distribution. (C and D) Immunohistochemical detection of pimonidazole and (E and F) merge images from the livers of (A, C and E) control rats and (B, D and F) rats with acute liver failure (ALF). Immunohistochemistry for (G and H) HIF1-α, (I and J) 4-HNE and (K and L) NOX4 in livers of (G, I and K) control rats and (H, J and L) rats with ALF. (M) Western blot analysis of NOX4. In the controls, TRITC-dextran was evenly distributed in the liver acinus lacking hypoxic areas (A and C). In the livers of rats with ALF, zone 1 was normoxic with sustained sinusoidal flow, but zone 3 was hypoxic with reduced sinusoidal flow (B, D and F). In the controls, all zones were predominantly negative for HIF1-α (G), 4-HNE (I) and NOX4 (K). In the ALF samples, zone 1 was mainly negative, and zone 3 positive for HIF1-α (H), 4-HNE (J) and NOX4 (L) (original magnification, ×200). In the livers of rats with ALF, NOX4 protein expression was increased compared with the controls (M) (^*^P≤0.05). P, portal vein; V, central vein. Zone 1, periportal zone; zone 2, intermediate zone; zone 3, pericentral zone.

On the basis of the results presented above, we hypothesized that hypoxia due to intrahepatic microcirculatory disorder may result in parenchymal oxidative stress and consequent apoptosis in zone 3 via an intrinsic pathway. In the control rats, whole liver tissue was negative for pimonidazole (normoxic conditions) (Fig. 4C). In the rats with ALF, the zone 1 parenchyma was predominantly negative for pimonidazole staining, whereas the zone 3 parenchyma was strongly positive (hypoxic conditions) (Fig. 4D). Hypoxia in zone 3 appeared to be induced by impaired intrahepatic microcirculation (Fig. 4F). An immunohistochemical detection of HIF1-α (Fig. 4G and H), 4-HNE (Fig. 4I and J) and NOX4 (Fig. 4K and L) was conducted. The whole liver tissue from the control rats was mostly negative, whereas the zone 3 parenchyma in the rats with ALF was positive for all proteins. The localization of HIF1-α, 4-HNE and NOX4 corresponded with that of pimonidazole. NOX4 protein expression was markedly increased in the rats with ALF compared with the control rats (Fig. 4M).

### Analysis of zonal differences in hepatocyte apoptosis

**Figure 5:**
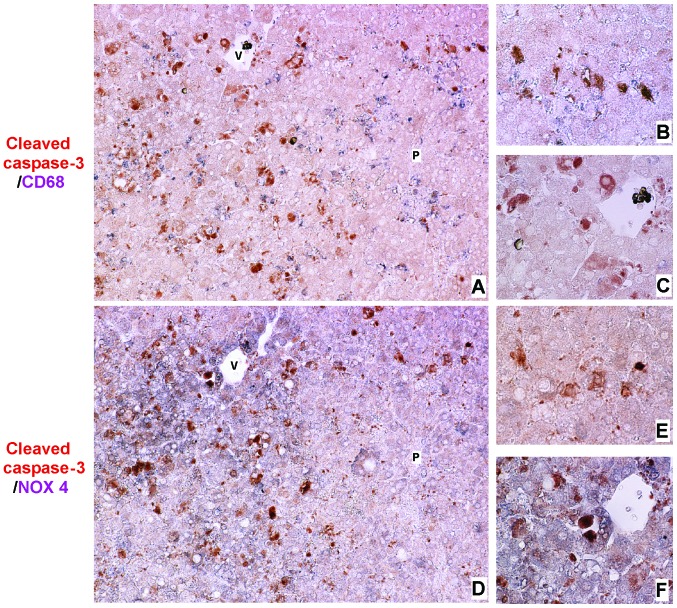
Simultaneous immunohistochemical detection of cleaved caspase-3 and CD68, and cleaved caspase-3 and nicotinamide adenine dinucleotide phosphate oxidase (NOX4) in livers of rats with acute liver failure (ALF). Immunohistochemical detection of cleaved caspase-3 (brown color) and CD68 (dark blue color) (A–C), and cleaved-caspase-3 (brown) and NOX4 (dark blue) (D–F) in livers of rats with ALF. The majority of apoptotic hepatocytes in zone 2 (B), and a few apoptotic hepatocytes in zone 3 (C) were localized adjacent to macrophages. A few apoptotic hepatocytes in zone 2 colocalized with NOX4 (E), and the majority of apoptotic hepatocytes in zone 3 were colocalized with NOX4 (F). Original magnification, ×200 (A and D), ×400 (B, C, E and F). P, portal vein; V, central vein. Zone 1, periportal zone; zone 2, intermediate zone; zone 3, pericentral zone.

The simultaneous immunohistochemical detection of cleaved caspase-3 and CD68 (Fig. 5A–C) revealed that the majority of the apoptotic hepatocytes in zone 2 were localized adjacent to infiltrated macrophages. Few apoptotic hepatocytes in zone 3 were similarly localized. The simultaneous immunohistochemical detection of cleaved caspase-3 and NOX4 (Fig. 5D–F) revealed that few apoptotic hepatocytes in zone 2 colocalized with NOX4, whereas the majority of apoptotic hepatocytes in zone 3 colocalized with NOX4.

## Discussion

In this study, we demonstrate that the intrahepatic microcirculatory structure can be visualized in formaldehyde-fixed paraffin-embedded (FFPE) liver sections from rats treated with TRITC-dextran. This method can be applied to both healthy and ALF livers stimulated with LPS/GalN. Previously, the administration of fluorescent dextran has been used to analyze tissue microcirculation under various conditions with the aim of demonstrating exceptional blood flow or extravasation. Vollmar *et al* (34) administered fluorescent dextran intravenously to rats with liver cirrhosis and observed its excretion to the lymph vessels. Sun *et al* (35) administered fluorescent dextran to rats with ischemia/reperfusion injury via the superior mesenteric artery to visualize extravasation at the intestinal villi. To our knowledge, no preceding study, however, has used this method to assess sinusoidal blood flow. This technically convenient method could be applied to evaluate other animal models mimicking liver diseases, such as graft-versus-host disease, sinusoidal obstruction syndrome and ischemia/reperfusion injury, which encompass intrahepatic microcirculatory disorder during their progression.

In our study, in the livers of rats with ALF, several spotty signals of TRITC-dextran were observed in zone 2 (Fig. 1D and F). These spotty signals seemed to represent a pooling of sinusoidal blood flow as a result of extravasation into the parenchyma. This likely indicates the disrupted integrity of sinusoidal endothelial cells (SECs), which is consistently described in previous studies involving animal models of ALF (36) and patients (37). Additionally, signals of TRITC-dextran in zone 3, the downstream region of sinusoidal flow, were markedly reduced (Fig. 1D, F and G). This is a reasonable result due to the destruction of the microcirculatory structure upstream of zone 3. To confirm whether these sinusoidal findings in ALF were connected to hepatic macrophage activation, we generated merged images comprising CD68 immunohistochemistry and TRITC-dextran fluorescent images.

Macrophage activation has been observed in patients with ALF (19,38) and is thought to be an important aspect of the pathogenesis of ALF (4,5). Nonetheless, the pathway from macrophage activation to massive hepatocyte death remains unclear. As sinusoidal fibrin deposition has been observed in patients with ALF (39,40), activated macrophages are thought to induce sinusoidal fibrin deposition and cause hepatic microcirculatory disorder, thereby resulting in parenchymal hypoxia and necrosis (13–18). An essential role of activated macrophages has been reported in parenchymal apoptosis through the secretion of death receptor ligands, such as Fas ligand and TNF-α (19,20). Evidence indicates that the same stimuli can result in either the necrosis or the apoptosis of hepatocytes (21). Bantel *et al* (41) showed that serum M30, which selectively recognizes a caspase cleaved neoepitope of cytokeratin 18, reflected hepatocyte apoptosis in patients with chronic hepatitis C. Rutherford *et al* (42,43) reported the findings of Bantel *et al* (41) as an effective predictor for the prognosis of ALF. Although the relative contribution of necrosis and apoptosis to ALF remains controversial (44), it is acceptable that a pathway triggered by macrophage activation that results in widespread hepatic hypoxia and subsequent massive hepatocyte death would exist, and that apoptosis would be involved in this process. Therefore, an investigation of the mechanisms of apoptosis in ALF may prove useful in the development of novel treatment strategies to protect liver cells from uncontrollable cell destruction.

In our rat model of ALF, CD68^+^ cells were localized around pooling TRITC-dextran in zone 2, indicating that infiltrated macrophages directly caused SEC destruction in that area (Fig. 2F). The number of infiltrated macrophages was most abundant in zone 2, followed by zone 3, and then zone 1 (Fig. 2G). The number of apoptotic hepatocytes was most abundant in zone 3, followed by zone 2 then zone 1 (Fig. 3E). The difference in the distribution of macrophages and apoptotic cells strongly suggests that the activation of an apoptotic pathway in zone 3 may be promoted by factors other than macrophages.

Apoptosis is induced by the sequential activation of caspases. This activation can be triggered by the activation of death receptors located on cell membranes (extrinsic pathway), and by cellular stress, i.e., endoplasmic reticulum stress or oxidative stress (intrinsic pathway) (21). Among the possible enzymatic systems contributing to the oxidative stress of hepatocytes (xanthine oxidoreductase, cyclooxygenase, endothelial nitric oxide synthases, cytochrome P450 monoxygenases and NOX) (45), NOX is a major source of cellular ROS (22–24). ROS generation by NOX has been considered to occur only in phagocytes. Six homologues of phagocyte catalytic NOX (NOX2) have been isolated: NOX1, NOX3, NOX4, NOX5, DUOX1 and DUOX2 (46). NOX4 has been shown to have unique characteristics compared with other NOX homologues. NOX4 requires no cytosolic component for ROS-producing activity and produces large amounts of ROS constitutively (47,48). NOX4 is expressed in fairly ubiquitous organs (49) and its expression levels are generally higher than those of other NOX homologues. NOX4 has therefore been suggested to be an inducible NOX (25). NOX4 has been shown to play a crucial role in the pathophysiology of cardiovascular diseases (23), diabetic complications (50) and fibrosis (51–53). In the liver, NOX4 is primarily expressed in hepatocytes, stellate cells and endothelial cells (54), and plays a pivotal role in the cellular senescence of hepatocytes (55), hepatitis C virus-induced ROS generation (56,57) and liver fibrosis (58–62).

Recently, NOX4 was found to be a target of HIF-1α, a master regulator of the cellular response to hypoxia (63), and therefore was suggested to be involved in cellular destruction in ALF. We hypothesized that intrahepatic microcirculatory disorder in the livers of rats with ALF may cause parenchymal hypoxia, and that the consequent activation of HIF1-α would upregulate NOX4, leading to the accumulation of oxidative stress and resultant hepatocyte apoptosis via an intrinsic pathway. In this study, zone 1 was almost normoxic and zone 3 was highly hypoxic (Fig. 4D). The hypoxic area was under impaired intrahepatic microcirculation (Fig. 4F) in the livers of rats with ALF. The results from immunohistochemistry for HIF1-α (Fig. 4G and H), 4-HNE (Fig. 4I and J) and NOX4 (Fig. 4K and L) suggested that their distributions were in accordance with those for pimonidazole. This indicates that oxidative stress may be induced by hypoxia-dependent NOX4 activation.

For a more precise evaluation of the localization of macrophages and NOX4-expressing cells in conjunction with that of apoptotic hepatocytes, we used simultaneous immunohistochemical staining. The majority of the apoptotic hepatocytes in zone 2 were localized adjacent to macrophages (Fig. 5B), but were not colocalized with NOX4-positive cells (Fig. 5E). The greatest portion of apoptotic hepatocytes in zone 3 was not localized adjacent to macrophages (Fig. 5C), but colocalized with NOX4-positive cells (Fig. 5F). These results indicated that hepatocyte apoptosis in zones 2 and 3 of our rat model of ALF was triggered mainly by activated macrophages and hypoxia, respectively.

To the best of our knowledge, ours is the first study to suggest that NOX4 upregulation may be involved in hepatocyte apoptosis during the progression of ALF. We recognize, however, that this study is based on histological observations only in one experimental animal model with no intervention. To date, NOX4-specific small-molecule inhibitors are not readily available (64,65), and further studies involving genetic interventions of NOX4 in an ALF model are required.

In conclusion, the intravenous injection of TRITC-dextran clearly revealed intrahepatic microcirculation in FFPE sections, and demonstrated intrahepatic microcirculatory disorder in the livers of rats with ALF. This method enables the simultaneous examination of microcirculation and immunohistochemistry to be conducted on the same liver sections, and is useful for investigating correlations between sinusoidal blood flow and other physiological factors. Zonal differences in hepatocyte apoptosis were revealed. The data from the present study suggest that hypoxia in zone 3 and intrahepatic microcirculatory disorder followed by HIF1-α upregulation may induce NOX4 upregulation, thus resulting in cellular oxidative stress.

## Abbreviations

ALF: acute liver failure
TRITC: tetramethylrhodamine isothiocyanate
LPS: lipopolysaccharide
GalN: D-galactosamine
TNF-α: tumor necrosis factor-α
NOX: nicotinamide adenine dinucleotide phosphate oxidase
ROS: reactive oxygen species
HIF1-α: hypoxia-inducible factor 1-α
4-HNE: 4-hydroxy-2-nonenal
HRP: horseradish peroxidase
PBS: phosphate-buffered saline
H&E: hematoxylin and eosin
DAB: 3,3′-diaminobenzidine tetrahydrochloride
ECL: enhanced chemiluminescence
FFPE: formaldehyde-fixed paraffin-embedded
SEC: sinusoidal endothelial cell
